# Differences in Pulmonary and Extra-Pulmonary Traits between Women and Men with Chronic Obstructive Pulmonary Disease

**DOI:** 10.3390/jcm11133680

**Published:** 2022-06-26

**Authors:** Sara Souto-Miranda, Alex J. van ‘t Hul, Anouk W. Vaes, Jeanine C. Antons, Remco S. Djamin, Daisy J. A. Janssen, Frits M. E. Franssen, Alda Marques, Martijn A. Spruit

**Affiliations:** 1 Department of Research and Development, Ciro, 6085 NM Horn, The Netherlands; anoukvaes@ciro-horn.nl (A.W.V.); daisyjanssen@ciro-horn.nl (D.J.A.J.); frits.franssen@ciro-horn.nl (F.M.E.F.); martijnspruit@ciro-horn.nl (M.A.S.); 2 Respiratory Research and Rehabilitation Laboratory (Lab3R) and Institute of Biomedicine (iBiMED), University of Aveiro, 3810-193 Aveiro, Portugal; amarques@ua.pt; 3 Department of Respiratory Medicine, Maastricht University Medical Centre, NUTRIM School of Nutrition and Translational Research in Metabolism, Faculty of Health, Medicine and Life Sciences, Maastricht University, 6229 ER Maastricht, The Netherlands; 4 Department of Respiratory Diseases, Radboud Institute for Health Sciences, Radboud University Medical Center, 6525 GA Nijmegen, The Netherlands; alex.vanthul@radboudumc.nl (A.J.v.‘t.H.); jeanine.antons@radboudumc.nl (J.C.A.); 5 Department of Respiratory Diseases, Amphia Hospital, 4818 CK Breda, The Netherlands; rdjamin@amphia.nl; 6 Department of Health Services Research, Care and Public Health Research Institute, Faculty of Health Medicine and Life Sciences, Maastricht University, 6226 NB Maastricht, The Netherlands

**Keywords:** COPD, sex-related differences, gender, treatable traits

## Abstract

Background: Evidence suggests sex-related differences in chronic obstructive pulmonary disease (COPD). Whether these differences are reflected in the prevalence of treatable traits remains unknown. Methods: Two samples of patients referred to secondary (n = 530) or tertiary care (n = 2012) were analyzed. Men and women were matched for age, forced expiratory volume in 1 s and body mass index. Sex-related differences were tested using *t*-tests, Mann-Whitney U, or chi-square tests. Results: Frequent exacerbations (30.5 vs. 19.7%), high cardiovascular risk (88.1 vs. 66.2%) and activity-related severe dyspnea (50.9 vs. 34.8%) were more prevalent in women in secondary care (*p* < 0.05). Severe hyperinflation (43.0 vs. 25.4%), limited diffusing capacity (79.6 vs. 70.1%), impaired mobility (44.0 vs. 28.7%), frequent exacerbations (66.8 vs. 57.4%), frequent hospitalizations (47.5 vs. 41.6%), severe activity-related dyspnea (89.1 vs. 85.0%), symptoms of anxiety (56.3 vs. 42.0%) and depression (50.3 vs. 44.8%), and poor health status (79.9 vs. 71.0%) were more prevalent in women in tertiary care (*p* < 0.05). Severe inspiratory muscle weakness (14.6 vs. 8.2%) and impaired exercise capacity (69.1 vs. 59.6%) were more prevalent among men (*p* < 0.05) in tertiary care. Conclusions: Sex-related differences were found, with most traits more prevalent and severe among women. Care providers should be aware of these differences to adjust treatment.

## 1. Introduction

In the past, chronic obstructive pulmonary disease (COPD) was considered a predominantly male disease. Nowadays, its prevalence is similar between women and men [1,2]. This may be explained by an increase in tobacco consumption by women in past decades, and due to environmental exposures (e.g., occupational dusts, household pollution), biological (i.e., sex hormones), genetic factors (i.e., higher predisposition to severe early-onset COPD) and anatomical differences (i.e., women have smaller airways) [3,4].

Despite the corresponding prevalence of COPD, sex-related bias in the diagnosis and treatment availability seems to exist, with women being less frequently diagnosed but better managed (i.e., women are more likely to receive treatments) [5,6]. In terms of clinical presentation, evidence suggests that women exhibit more frequently the chronic bronchitis phenotype [7], higher levels of dyspnea [8] and symptoms of anxiety and depression [9], and less exercise tolerance when compared to men [8]. However, conflicting data exist in terms of sex-related differences on health-related quality of life, number of exacerbations and hospital admissions [3,7,8,10]. Little is also known about sex-related differences in other important features such as body composition, physical activity, and fatigue. Furthermore, studies have lacked matching of female and male patients with COPD for important confounders such as age, forced expiratory volume in one second (FEV_1_), and body mass index (BMI) [4].

Additionally, whether these differences are reflected in the prevalence of treatable traits (i.e., traits that are clinically relevant, identifiable, measurable, and treatable) [11] requires further investigation. This is of clinical importance, as recognizing treatable traits early in the disease trajectory may help clinicians to timely personalize interventions and guide treatment more efficiently [11].

Thus, we aimed to explore sex-related differences in pulmonary, extra-pulmonary, and behavioral traits of patients with COPD referred to secondary or tertiary care.

## 2. Materials and Methods

This was an observational retrospective study with two samples of patients with COPD referred to: (1) the first-ever secondary care outpatient consultation in Amphia Hospital in Breda, in the Radboud University Medical Centre in Nijmegen or in the Bernhoven Hospital in Uden between April 2013 and June 2017; or (2) to a tertiary center of care (Ciro) between January 2013 and February 2020 (all in The Netherlands). The Medical Ethical Committee of the Radboudumc (ref. 2016–2603); and the Maastricht University Medical Center (ref. 8552) approved these retrospective studies. Participants were subjected to usual care, hence, both datasets were not considered to fall within the remit of the Medical Research Involving Human Subjects Act (WMO).

This study used two existing datasets extracted from medical records and is therefore limited in terms of the information available (i.e., medications and comorbidities were not captured). Inclusion criteria were patients with a primary diagnosis of COPD confirmed by spirometry, with a post-bronchodilator FEV_1_/forced vital capacity ratio (FEV1/FVC) < 0.70. Participants were excluded if they had a COPD exacerbation in the previous month and physical impairments that precluded valid assessments (e.g., wheelchair).

Patients from both sexes were automatically matched for age (within 3 years), FEV_1_% predicted (within 5%) and BMI (within 4 kg/m^2^) using the case-control matching procedure of SPSS Statistics (v24, IBM, Armonk, NY, USA). These interval values were chosen as they maximized sample size without significant differences between women and men for these variables.

### 2.1. Data Collection

Age and sex, and a comprehensive clinical assessment was performed in both samples consisting of smoking history, number of exacerbations and hospitalizations in the last 12 months, BMI (computed from weight in kilograms and squared height in meters), spirometry [12], 6-min walk test (6MWT) where the best of 2 tests was considered [13], and mMRC dyspnea grade [14]. Patients were classified in terms of disease severity (grades 1–4) and symptoms and exacerbation risk (ABCD assessment tool) with the modified medical research council dyspnea scale (mMRC) and number of exacerbations and hospitalizations in the previous 12 months, according to the Global initiative for chronic obstructive lung disease (GOLD) [15].

Additional measurements for each sample are listed in Table S1 [see Supplementary Materials] and consisted of use of long-term oxygen therapy and of a walking aid; waist circumference (to define the cardiovascular risk—probability of having a cardiovascular event/problem, e.g., atherosclerosis [16]); dual-energy X-ray absorptiometry (DEXA) [17], where lean mass index (LMI) was calculated as (fat-free mass–bone mass content)/height^2^ [18]; whole-body plethysmography, single breath carbon monoxide diffusing capacity (DLCO) and maximum inspiratory mouth pressure as recommended [19,20,21]; maximal cycle cardiopulmonary exercise test (CPET) [22], and a constant work rate test (CWRT) [23]; 1 maximum repetition (1RM) of leg press and leg extension; number of steps with a uniaxial (Digiwalker SW-200; Yamax Corporation, Tokyo, Japan) or a triaxial accelerometer (DynaPort MoveMonitor, McRoberts, The Hague, The Netherlands) [24]; the checklist of individual strength-fatigue (CIS-F) [25]; the hospital anxiety and depression scale (HADS) [26]; the clinical COPD questionnaire (CCQ) [27], and the COPD assessment test (CAT) [28].

Details of the assessments for each sample have been published elsewhere [29,30,31,32].

Treatable traits were defined through previously established cut-offs of each variable and are presented in Table 1.

### 2.2. Statistical Analysis

Descriptive statistics were computed for the total samples and for each sex group. Normally distributed variables were presented as mean ± standard deviation, whereas non-normal distributed variables were presented as median [interquartile range] and categorical variables as frequencies.

Differences between women and men with COPD were tested using independent samples *t*-tests or Mann–Whitney U-tests for continuous variables, and chi-square tests for categorical variables.

A *p*-value of <0.05 was set for statistical significance. All analyses were performed using SPSS Statistics (v24, IBM, Armonk, NY, USA).

## 3. Results

**Figure 1:**
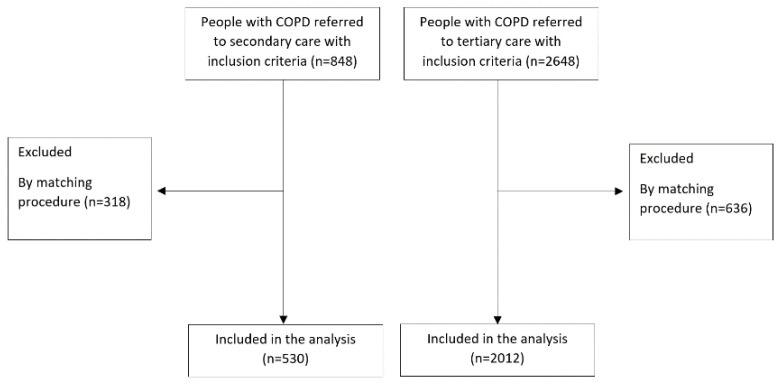
Flowchart of study for both samples.

In total, 848 and 2648 participants from secondary and tertiary care met the inclusion criteria, respectively. With the matching procedure, 318 and 636 patients were excluded, and therefore 530 and 2012 patients from secondary and tertiary care were included, respectively (Figure 1).

Patients from secondary care were younger, had higher FEV_1_, higher FEV_1_/FVC, less frequent exacerbations and hospitalizations <12 months, were less frequently underweight/obese, had less dyspnea and symptom burden according to GOLD, and a higher proportion were active smokers compared to patients referred to tertiary care. A total of 18 treatable traits were found; 11 traits were found for the secondary care sample and 15 for the tertiary care sample, with 8 traits in common for both samples.

### 3.1. Pulmonary Traits

**Figure 2:**
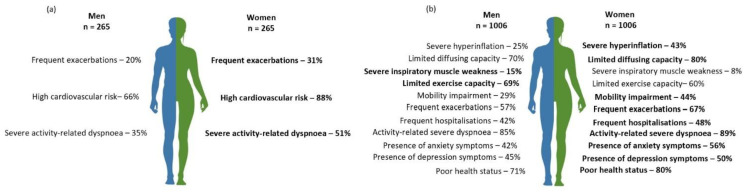
Prevalence of pulmonary and extra-pulmonary traits (according to the cut-offs of Table 1) that are different between women and men with chronic obstructive pulmonary disease (COPD) referred to (**a**) secondary care (n = 530) or (**b**) tertiary care (n = 2012); (*p* < 0.05). Blue represents men and green represents women. Bold represents where (gender) the trait is more prevalent. There were 11 and 15 treatable traits assessed in secondary and tertiary care, respectively.

In the secondary care sample, a higher proportion of women presented frequent exacerbations (*p* = 0.008). No other significant differences in pulmonary traits were found (Table 2 and Figure 2).

In tertiary care, women presented higher intrathoracic gas volume, residual volume (RV), total lung capacity (TLC), RV/TLC, and lower DLCO than men (all *p* < 0.001). A higher proportion of women presented severe static lung hyperinflation (*p* < 0.001) and limited diffusing capacity for carbon monoxide (both *p* < 0.001). Also, long-term oxygen therapy was used more frequently in women than men (*p* < 0.001). When considering reference values, men showed worse inspiratory muscle strength, and more frequently severe inspiratory muscle weakness than women (*p* < 0.001). The highest number of acute exacerbations (*p* < 0.001) and hospitalizations (*p* = 0.021) in the previous 12 months was found for women, with a larger proportion of women than men having frequent exacerbations (*p* < 0.001), and hospitalizations (*p* = 0.008). No other significant differences in pulmonary traits were found (Table 2 and Figure 2).

### 3.2. Extra-Pulmonary Traits

Women in secondary care had higher mMRC scores (*p* < 0.001), and more frequently activity-related severe dyspnea than men (*p* = 0.006). Women also had lower waist circumference than men, but higher cardiovascular risk considering the cut-off (*p* < 0.001).

In tertiary care, a higher proportion of women had activity-related severe dyspnea (*p* = 0.006) and exhibited more frequently anxiety (*p* < 0.001) and depression symptoms than men (*p* = 0.015). Women had higher body fat mass (*p* = 0.002), lower bone mass content (*p* < 0.001) and lower fat-free mass (*p* < 0.001), which translated into a lower LMI than men (*p* < 0.001). The use of a walking aid was also more frequently observed in women than men (*p* < 0.001.). Considering the % predicted values, exercise capacity was lower in men (*p* < 0.001), with more men than women showing limited maximal and functional exercise capacity (*p* < 0.001). Significant differences were also found for quadriceps muscle strength with women presenting less strength than men (*p* < 0.001). In terms of health status, women had higher (worse) CAT scores than men with more frequently poor health status than men (*p* < 0.001). No other significant differences in extra-pulmonary traits were found (Table 3 and Figure 2).

### 3.3. Behavioral Traits

In secondary care, the proportion of active smokers or physically inactive patients was similar between women and men.

In tertiary care, men were heavier smokers with higher pack-years than women (*p* < 0.001) but with no differences in the number of active smokers (women 24.6% vs. men 24.5%, *p* = 0.346) (Table 3).

## 4. Discussion

This study shows the presence of sex-related differences in pulmonary and extra-pulmonary treatable traits in patients with COPD, after matching for age, FEV_1_% predicted and BMI. Some of these sex-related differences (e.g., higher dyspnea in women) were already present in patients with COPD who had their first-ever secondary care outpatient appointment. A recent study has also found higher symptom burden in women than men with COPD, even in younger populations [48]. Healthcare providers should be aware that women may have worse outcomes from the start of the disease path, so they can recognize their needs and adjust treatment early. Therefore, these differences should be considered in clinical practice when planning personalized interventions.

### 4.1. Pulmonary Traits

With the same degree of airway obstruction, women have more static hyperinflation of the lungs, a common result of emphysema [49], which can impact other outcomes, namely dyspnea during activities/exercise [50]. This finding, as well as the observed limited diffusing capacity of women compared to men, is consistent with a recent study that found RV/TLC to be higher and DLCO to be lower in women than men [10]. The participants of this study were not submitted to lung volume reduction surgery prior to entering the study, but it was not possible to distinguish risk factors other than smoking, e.g., occupational exposure to dusts, or confirm phenotypes (emphysema vs chronic bronchitis), which could explain these differences, hence future studies are needed.

This study suggests that men with COPD have worse respiratory muscle function, and present severe inspiratory muscle weakness more frequently than women with this disease. Since more women than men presented severe hyperinflation which is a determinant of poor inspiratory muscle strength [51], this result was unexpected. This finding is however based on reference values. It is possible that the equation used by Black and Hyatt [36] is not the most suitable for the Dutch population, which has been observed in other countries [52].

Conflicting evidence exists in the literature regarding sex-related differences in exacerbations, and hospitalizations [3,7,8,10]. In the present study we found women to have a higher number of exacerbations in both samples (less symptomatic and more functional and more symptomatic and less functional), and therefore we believe these results are a good representation of the COPD spectrum. We only observed sex-differences in hospitalizations of patients referred to tertiary care, which might be explained by the fact that patients in secondary care were having their first-ever pulmonology appointment and therefore were in the early stages of COPD, with none to few hospitalizations. Evidence has suggested women with COPD to be more extensively managed than men [6], thus it is possible that besides physiological differences, women also seek medical care more frequently or sooner. Considering these sex-related differences, these treatable traits should be frequently assessed, especially in women, and when detected should prompt healthcare providers to refer patients to self-management and pulmonary rehabilitation interventions [53,54,55].

### 4.2. Extra-Pulmonary Traits

Higher levels of dyspnea, anxiety, and depression in women compared to men were found and are consistent with recent literature [6,8,10,56]. This disparity between women and men in symptoms has been thought to exist because it is more culturally acceptable for women than men to express their feelings [57]. Nevertheless, there are differences in other characteristics such as lung function hence, a complex interplay of several factors (i.e., physiological, psychological, social) might be more plausible and should be further explored. These treatable traits should be screened, and when detected patients should be guided through the most appropriate treatment (e.g., pulmonary rehabilitation, cognitive behavioral therapy, or palliative symptom management) [58,59,60,61].

Our study also found women to have more frequently mobility impairments (i.e., using a walking aid) than men, which might be due to women reporting more frequently mobility problems and receiving medical support more frequently than men [6,62], or due to a higher prevalence of musculoskeletal comorbidities than men [63]. Additionally, we also observed a higher percentage of women presenting high cardiovascular risk, which contradicts evidence showing that although women have a poorer prognosis, the prevalence of cardiovascular disease is lower than in men [56,64]. Due to the lack of data available, the impact of comorbidities and medication on these differences in mobility impairments (e.g., due to osteoporosis, use of corticosteroids) and on the actual prevalence of cardiovascular disease is unclear.

In terms of health status/health-related quality of life, it is still not clear if sex-related differences exist [3,7]. We found health status and functional exercise capacity to be different between women and men only in patients referred to tertiary care. This might be explained by sample-specific differences, i.e., patients referred to secondary care were less symptomatic, had better lung function, less exacerbations, and better functional capacity, suggesting that sex-related differences might be more prominent as the disease progresses. Nevertheless, the high prevalence of these treatable traits among patients with COPD requires attention and should prompt clinicians to refer patients to pulmonary rehabilitation [55,58].

### 4.3. Behavioral Traits

We found no sex-related differences in the number of active smokers and with physical inactivity. However, physical activity was only assessed in the secondary care sample, and similarly to other outcomes, it is possible that differences could occur later in the disease trajectory. Hence, future studies should explore sex-differences in physical activity and other behavioral traits such as social support or self-management skills, as these could aid personalizing interventions [11,65].

### 4.4. Strengths and Limitations

Future studies should investigate the impact of the identified differences in treatable traits of men and women with COPD on the response to different interventions.

Sex is biological and determined at conception and gender is a social construct [4]. Whilst some of our traits are related to sex differences (e.g., physiological measures), others are dependent on gender (e.g., patient-reported outcomes). Our samples were binary, and therefore these differences should be further explored in non-binary populations.

This study included a good sample size, was matched for important confounders (age, FEV_1_, BMI), and had patients with COPD from two different samples with different disease states, which is important for the external validity of findings. Nevertheless, some limitations exist. Although we had a large sample size, it is possible that these results are restricted to Caucasian and European populations. Moreover, due to the lack of published cut-offs, some treatable traits, such as peripheral muscle weakness, were not possible to determine. Additionally, due to the inherent limitations of a retrospective study, many other treatable traits and characteristics such as comorbidities, medication use, systemic inflammation, hypoxemia, and blood eosinophilia were not possible to determine as these data were not available from the medical records extracted. Data on the type of medication and comorbidities would be particularly valuable to understand if sex-differences were related with other concomitant diseases. Hence, future studies should explore sex-related differences in other missing but important treatable traits and possible explanations for these disparities between women and men with COPD.

## 5. Conclusions

Sex-related differences were found in pulmonary and extra-pulmonary traits of patients with COPD, with most traits being more prevalent and severe among women than men. Care providers should be aware of these differences to early detect patients’ needs and adjust treatment.

## Figures and Tables

**Table 1:** Cut-off values used to identify pulmonary, extra-pulmonary, and behavioural traits in people with chronic obstructive pulmonary disease.

Trait	Cut-Off	Reference(s)
**Pulmonary traits**		
Severe hyperinflation	RV/TLC ≥ 0.58	[33]
Limited diffusing capacity	DLCO < 60% predicted	[34]
Severe inspiratory muscle weakness	Pimax < 50% predicted	[35,36]
Frequent exacerbations	≥2 previous year	[37]
Frequent hospital admission	≥1 previous year	[37]
**Extra-pulmonary traits**		
***Extra-pulmonary-symptoms***		
Severe activity-related dyspnea	mMRC ≥ 2	[38]
Severe fatigue	CIS-F ≥ 36 points	[39]
Symptoms of anxiety	HADS ≥ 8 points	[40]
Symptoms of depression	HADS ≥ 8 points	[40]
***Extra-pulmonary–health status***		
Poor health status	CAT ≥ 18 points CCQ ≥ 1.9 points	[41] [41]
***Extra-pulmonary-physical***		
Underweight Obese	BMI < 21 kg/m^2^ BMI > 30 kg/m^2^	[42]
Low muscle mass	LMI < 10th percentile	[18]
High cardiovascular risk	Waist circumference ≥ 80 cm in women and ≥ 94 cm in men	[43]
Limited exercise capacity	6MWD < 70% predicted CPET workmax < 70% predicted	[31] [44] [45]
**Behavioral traits**		
Low physical activity	<5000 steps/day	[46]
Current smoking	N.A.	N.A.

RV/TLC: Residual volume/Total lung capacity; DLCO: Diffusing capacity of carbon monoxide; Pimax: Maximal inspiratory mouth pressure; mMRC: Modified medical research council dyspnea scale; CIS-F: Checklist of individual strength–fatigue subscale; HADS: The hospital anxiety and depression scale; CAT: COPD assessment test; CCQ: Clinical COPD questionnaire; BMI: Body mass index; LMI: Lean mass index; 6MWD: Six-minute walking distance; CPET: Cardiopulmonary exercise testing; N.A.: Not applicable.

**Table 2:** Sociodemographic characteristics and pulmonary traits of female and male patients with chronic obstructive pulmonary disease (COPD) and comparison between secondary care (n = 530) and tertiary care samples (n = 2012).

	Patients Referred to Secondary Care				Patients Referred to Tertiary Care				
	Total Sample (n = 530)	Female (n = 265; 50%)	Male (n = 265; 50%)	*p*-Value	Total Sample (n = 2012)	Female (n = 1006; 50%)	Male (n = 1006; 50%)	*p*-Value	Between Total Samples *p*-Value
Age, years	63.3 ± 8.4	63.2 ± 8.4	63.3 ± 8.4	0.885	65.7 ± 7.9	65.6 ± 7.9	65.9 ± 8.0	0.330	<0.001 *
40–49, n (%)	21 (4.0)	11 (4.1)	10 (3.8)	0.820	49 (2.4)	21 (2.1)	28 (2.8)	0.738	<0.001 *
50–59, n (%)	160 (30.2)	82 (30.9)	78 (29.4)		445 (22.1)	230 (22.9)	215 (21.4)		
60–69, n (%)	215 (40.6)	103 (38.9)	112 (42.3)		894 (44.4)	451 (44.8)	443 (44.0)		
70–79, n (%)	119 (22.5)	63 (23.8)	56 (21.1)		571 (28.4)	278 (27.6)	293 (29.1)		
80–89, n (%)	15 (2.8)	6 (2.3)	9 (3.4)		53 (2.6)	26 (2.6)	27 (2.7)		
**Pulmonary Traits**									
FEV_1_% predicted	55.2 [43.1–68.3]	55.0 [43.0–68.3]	55.7 [43.3–67.7]	0.999	43.6 [32.1–59.5]	43.6 [32.4–59.8]	43.5 [31.6–59.2]	0.733	<0.001 *
GOLD 1, n (%)	51 (9.6)	23 (8.7)	28 (10.5)	0.649	118 (6.0)	55 (5.6)	64 (6.4)	0.415	<0.001 *
GOLD 2, n (%)	273 (51.5)	139 (52.5)	134 (50.5)		639 (32.2)	324 (32.8)	315 (31.6)		
GOLD 3, n (%)	197 (37.2)	100 (37.7)	97 (36.7)		811 (40.9)	415 (42.0)	396 (39.8)		
GOLD 4, n (%)	9 (1.7)	3 (1.1)	6 (2.3)		416 (21.0)	195 (19.7)	221 (22.2)		
FVC, % predicted	92.1 ± 17.1	91.8 ± 16.6	92.3 ± 17.6	0.755	94.7 ± 21.4	94.1 ± 21.6	95.3 ± 21.2	0.200	0.003 *
FEV_1_/FVC	48.6 ± 11.9	49.8 ± 12.3	47.5 ± 11.4	0.024 *	38.5 ± 12.5	39.1 ± 11.8	37.9 ± 13.1	0.003 *	<0.001 *
ITGV, % predicted	N.A.	N.A.	N.A.	N.A.	148.4 [121.7–175.7]	152.8 [129.8–180.6]	142.9 [114.1–170.7]	<0.001 *	N.A.
ERV, % predicted	N.A.	N.A.	N.A.	N.A.	111.7 [84.8–141.1]	109.7 [84.1–140.0]	114.6 [86.1–142.2]	0.122	N.A.
RV, % predicted	N.A.	N.A.	N.A.	N.A.	158.9 [123.8–197.3]	166.2 [134.3–202.4]	151.7 [115.5–190.0]	<0.001 *	N.A.
TLC, % predicted	N.A.	N.A.	N.A.	N.A.	117.6 [104.8–130.0]	123.1 [110.2–134.3]	112.7 [100.2–125.1]	<0.001 *	N.A.
RV/TLC, %	N.A.	N.A.	N.A.	N.A.	53.2 ± 11.5	56.2 ± 11.1	50.3 ± 11.1	<0.001 *	N.A.
**RV/TLC ≥ 0.58, n (%)**	N.A.	N.A.	N.A.	N.A.	663 (34.1)	414 (43.0)	249 (25.4)	<0.001 *	N.A.
DLCO, % predicted	N.A.	N.A.	N.A.	N.A.	47.8 [37.8–60.3]	46.0 [37.1–57.2]	49.7 [38.6–63.4]	<0.001 *	N.A.
**DLCO < 60%**	N.A.	N.A.	N.A.	N.A.	1383 (74.7)	712 (79.6)	671 (70.1)	<0.001 *	N.A.
Kco, % predicted	N.A.	N.A.	N.A.	N.A.	60.2 [48.7–78.1]	57.1 [47.3–73.7]	63.7 [50.6–83.0]	<0.001 *	N.A.
Pimax, cmH_2_O	N.A.	N.A.	N.A.	N.A.	68.6 ± 21.7	61.4 ± 19.2	75.9 ± 21.6	<0.001 *	N.A.
Pimax, % predicted	N.A.	N.A.	N.A.	N.A.	78.9 ± 24.4	86.9 ± 26.1	71.0 ± 19.6	<0.001 *	N.A.
**Pimax < 50% predicted**	N.A.	N.A.	N.A.	N.A.	226 (11.4)	81 (8.2)	145 (14.6)	<0.001 *	N.A.
AECOPD past 12 months, n ^a^	0.0 [0.0–1.0]	1.0 [0.0–2.0]	0.0 [0.0–1.0]	0.063	2.0 [1.0–4.0]	2.0 [1.0–4.0]	2.0 [1.0–4.0]	<0.001 *	<0.001 *
**≥2 AECOPD, n (%) ^a^**	114 (25.0)	68 (30.5)	46 (19.7)	0.008 *	1241 (62.1)	669 (66.8)	572 (57.4)	<0.001 *	<0.001 *
Hospitalisations due to COPD previous 12 months, n ^a^	0.0 [0.0–0.0]	0.0 [0.0–0.0]	0.0 [0.0–0.0]	0.086	0.0 [0.0–1.0]	0.0 [0.0–1.0]	0.0 [0.0–1.0]	0.021 *	<0.001 *
**≥1 hospitalisations, n (%) ^a^**	31 (7.3)	18 (8.8)	13 (6.0)	0.267	890 (44.5)	475 (47.5)	415 (41.6)	0.008 *	<0.001 *
GOLD groups (A-D), n (%)									
GOLD A	42 (12.6)	71 (40.3)	104 (53.1)	0.031 *	24 (1.2)	7 (0.7)	17 (1.7)	<0.001 *	<0.001 *
GOLD B	110 (32.9)	49 (27.9)	51 (26.0)		301 (15.0)	130 (12.9)	171 (17.0)		
GOLD C	30 (9.0)	22 (12.5)	21 (10.7)		53 (2.6)	18 (1.8)	35 (3.5)		
GOLD D	152 (45.5)	34 (19.3)	20 (10.2)		1631 (81.2)	850 (84.6)	781 (77.8)		
LTOT, n (%)	N.A.	N.A.	N.A.	N.A.	412 (21.0)	233 (23.8)	179 (18.3)	<0.001 *	N.A.

* Statistically significant. ^a^ The secondary care sample had more than 10% missing data for the variables number of acute exacerbations and hospitalisations, waist circumference, modified medical research council dyspnoea scale (mMRC), and the checklist of individual strength–fatigue subscale (CIS-F). The tertiary care sample had less than 10% of missing data [47] for all considered variables. FEV_1_: Forced expiratory volume in 1 s; GOLD: Global initiative for chronic obstructive lung disease; FVC: forced vital capacity; ITGV: Intrathoracic gas volume; ERV: Expiratory reserve volume; RV: Residual volume; TLC: Total lung capacity; DLCO: Diffusing capacity for carbon monoxide; Kco: Carbon monoxide transfer coefficient; Pimax: Maximal inspiratory mouth pressure; cmH_2_O: Centimetre of water; AECOPD: Acute exacerbations of COPD; LTOT: Long-term oxygen therapy. N.A.: Not assessed.

**Table 3:** Extra-pulmonary and behavioural traits of female and male patients with chronic obstructive pulmonary disease (COPD) and comparison between secondary care (n = 530) and tertiary care samples (n = 2012).

	Patients Referred to Secondary Care				Patients Referred to Tertiary Care				
	Total Sample (n = 530)	Female (n = 265; 50%)	Male (n = 265; 50%)	*p*-Value	Total Sample (n = 2012)	Female (n = 1006; 50%)	Male (n = 1006; 50%)	*p*-Value	Between Total Samples *p*-Value
**Extra-Pulmonary Traits-Symptoms**									
mMRC, score ^a^	1.0 [0.0–2.0]	2.0 [1.0–2.0]	1.0 [0.0–2.0]	<0.001 *	2.0 [2.0–3.0]	2.0 [2.0–3.0]	2.0 [2.0–3.0]	0.006 *	<0.001 *
mMRC ≥2 points ^a^	197 (42.7)	116 (50.9)	81 (34.8)	<0.001 *	1737 (87.0)	889 (89.1)	848 (85.0)	0.006 *	<0.001 *
CIS-F, score ^a^	37.0 [27.0–47.0]	37.0 [28.0–47.0]	37.0 [26.0–47.0]	0.604	N.A.	N.A.	N.A.	N.A.	N.A.
CIS-F score < 36	182 (47.4)	86 (46.7)	96 (48.0)	0.805	N.A.	N.A.	N.A.	N.A.	N.A.
CIS-F score ≥ 36	202 (52.6)	98 (53.3)	104 (52.0)		N.A.	N.A.	N.A.	N.A.	N.A.
HADS, anxiety score	N.A.	N.A.	N.A.	N.A.	7.0 [4.0–11.0]	8.0 [5.0–11.0]	7.0 [4.0–10.0]	<0.001 *	N.A.
HADS, anxiety score ≥ 8 points	N.A.	N.A.	N.A.	N.A.	941 (49.2)	544 (56.3)	397 (42.0)	<0.001 *	N.A.
HADS, depression score	N.A.	N.A.	N.A.	N.A.	7.0 [4.0–10.0]	8.0 [4.0–10.0]	7.0 [4.0–10.0]	0.002 *	N.A.
HADS, depression score ≥ 8 points	N.A.	N.A.	N.A.	N.A.	909 (47.6)	486 (50.3)	423 (44.8)	0.015 *	N.A.
**Extra-Pulmonary Traits–Health Status**									
CAT, score	N.A.	N.A.	N.A.	N.A.	22.0 [18.0–26.0]	23.0 [19.0–27.0]	21.0 [16.0–25.0]	<0.001 *	N.A.
CAT ≥18 points	N.A.	N.A.	N.A.	N.A.	1448 (75.5)	773 (79.9)	675 (71.0)	<0.001 *	N.A.
CCQ, score									
CCQ symptoms	2.5 [1.5–3.3]	2.3 [1.5–3.3]	2.5 [1.5–3.3]	0.232	N.A.	N.A.	N.A.	N.A.	N.A.
CCQ functional state	1.8 [1.0–3.0]	1.8 [1.0–3.2]	1.8 [1.0–3.0]	0.387	N.A.	N.A.	N.A.	N.A.	N.A.
CCQ mental state	1.0 [0.0–2.0]	1.0 [0.0–2.5]	1.0 [0.0–2.0]	0.269	N.A.	N.A.	N.A.	N.A.	N.A.
CCQ, total score	1.8 [1.2–2.9]	1.9 [1.2–2.9]	1.8 [1.2–2.9]	0.515	N.A.	N.A.	N.A.	N.A.	N.A.
CCQ, total score ≥ 1.9	237 (49.5)	123 (51.5)	114 (47.5)	0.386	N.A.	N.A.	N.A.	N.A.	N.A.
**Extra-Pulmonary Traits–Physical**									
BMI, Kg/m^2^	25.2 ± 4.6	25.2 ± 4.6	25.2 ± 4.5	0.901	25.4 [21.8–29.4]	25.1 [21.5–29.3]	25.6 [22.1–29.6]	0.078	0.049 *
BMI < 21, n (%)	98 (18.5)	51 (19.2)	47 (17.7)	0.811	409 (20.3)	219 (21.8)	190 (18.9)	0.275	<0.001 *
BMI > 30, n (%)	77 (14.5)	40 (15.1)	37 (14.0)		451 (22.4)	222 (22.1)	229 (22.8)		
Total body fat, Kg	N.A.	N.A.	N.A.	N.A.	25.4 [18.2–33.3]	26.0 [18.7–34.6]	24.8 [17.7–31.9]	0.002 *	N.A.
Bone mass content, Kg	N.A.	N.A.	N.A.	N.A.	23.1 [19.3–27.4]	19.4 [17.4–21.8]	27.2 [24.5–30.4]	<0.001 *	N.A.
Fat-free mass, Kg	N.A.	N.A.	N.A.	N.A.	45.8 [39.0–53.5]	39.2 [35.8–43.1]	53.2 [48.3–58.7]	<0.001 *	N.A.
LMI, Kg/m^2^	N.A.	N.A.	N.A.	N.A.	15.4 [13.8–17.3]	14.2 [13.1–15.5]	16.8 [15.4–18.5]	<0.001 *	N.A.
LMI < 10th percentile, n (%)	N.A.	N.A.	N.A.	N.A.	524 (26.0)	255 (25.3)	269 (26.7)	0.477	N.A.
Waist circumference, cm ^a^	96.6 ± 12.9	93.0 ± 12.0	99.9 ± 12.9	<0.001 *	N.A.	N.A.	N.A.	N.A.	N.A.
Waist circumference, ≥80 cm women, ≥94 cm men	324 (76.6)	177 (88.1)	147 (66.2)	<0.001 *	N.A.	N.A.	N.A.	N.A.	N.A.
Use of walking aid, n (%)	N.A.	N.A.	N.A.	N.A.	723 (36.4)	437 (44.0)	286 (28.7)	<0.001 *	N.A.
6MWD, m	450.0 [372.0–512.0]	420.0 [355.0–491.0]	479.0 [400.0–530.0]	<0.001 *	395.0 [313.5–467.0]	375.0 [295.0–441.0]	415.0 [339.5–486.5]	<0.001 *	<0.001 *
6MWD, % predicted	69.0 [60.0–77.7]	69.5 [61.4–76.6]	68.7 [78.6]	0.811	64.0 [51.0–74.0]	66.0 [53.0–76.0]	62.0 [50.0–72.0]	<0.001 *	<0.001 *
6MWD < 70% predicted	284 (53.6)	142 (53.6)	142 (53.6)	1.000	1276 (64.4)	589 (59.6)	687 (69.1)	<0.001 *	<0.001 *
CPET Workmax, Watts	N.A.	N.A.	N.A.	N.A.	60.0 [43.0–81.0]	51.0 [38.0–67.0]	70.0 [51.0–93.5]	<0.001 *	N.A.
CPET Workmax, % predicted	N.A.	N.A.	N.A.	N.A.	45.0 [34.0–58.0]	50.0 [38.0–64.0]	41.0 [31.0–52.0]	<0.001 *	N.A.
Workmax, <70% predicted	N.A.	N.A.	N.A.	N.A.	1627 (88.1)	752 (82.5)	875 (93.5)	<0.001 *	N.A.
CWRT, Workmax, Wattsa	N.A.	N.A.	N.A.	N.A.	46.0 [33.0–61.0]	39.0 [29.0–50.0]	52.0 [29.0–70.0]	<0.001 *	N.A.
CWRT time cycled, s ^a^	N.A.	N.A.	N.A.	N.A.	215.0 [160.0–303.0]	200.0 [152.0–274.0]	235.0 [169.0–335.0]	<0.001 *	N.A.
1 RM Leg extension, Kg	N.A.	N.A.	N.A.	N.A.	27.5 [20.0–37.5]	22.5 [15.0–30.0]	35.0 [25.0–45.0]	<0.001 *	N.A.
1 RM Leg press, Kg	N.A.	N.A.	N.A.	N.A.	70.0 [50.0–100.0]	50.0 [30.0–70.0]	90.0 [60.0–120.0]	<0.001 *	N.A.
**Behavioural Traits**									
Smoking status, n (%)									
Former smoker	90 (17.5)	50 (19.5)	40 (15.4)	0.187	1442 (72.0)	726 (72.5)	716 (71.4)	0.346	<0.001 *
Current smoker	229 (44.5)	118 (46.1)	111 (42.9)		492 (24.6)	246 (24.6)	246 (24.5)		
Never smoker	196 (38.1)	88 (34.4)	108 (41.7)		70 (3.5)	29 (2.8)	41 (4.0)		
Pack-years, n	N.A.	N.A.	N.A.	N.A.	40.0 [30.0–52.0]	40.0 [28.0–50.0]	44.0 [30.0–60.0]	<0.001 *	N.A.
Steps per day, n	5008.0 [3043.67–7433.80]	4795.0 [2842.50–7200.0]	5118.67 [3138.0–7782.0]	0.159	N.A.	N.A.	N.A.	N.A.	N.A.
Steps per day < 5000	264 (49.8)	138 (52.1)	126 (47.5)	0.297	N.A.	N.A.	N.A.	N.A.	N.A.

* Statistically significant. ^a^ The secondary care sample had more than 10% missing data for the variables number of acute exacerbations and hospitalisations, waist circumference, modified medical research council dyspnoea scale (mMRC), and the checklist of individual strength–fatigue subscale (CIS-F). The tertiary care sample had less than 10% of missing data [47] for all considered variables. mMRC: Modified medical research council dyspnoea scale; CIS-F: Checklist of individual strength-fatigue scale; HADS: The hospital anxiety and depression scale; CAT: COPD assessment test; CCQ: Clinical COPD questionnaire; BMI: Body mass index; LMI: Lean mass index; 6MWD: Six-minute walking distance; CPET: Cardiopulmonary exercise testing; CWRT: Constant work rate test; 1RM: 1 maximum repetition. N.A.: Not assessed.

## Data Availability

The data presented in this study are available in the article and Supplementary Material.

## Supplementary Materials

The following supporting information can be downloaded at: https://www.mdpi.com/article/10.3390/jcm11133680/s1, Table S1: Differences in data collected from patients with chronic obstructive pulmonary disease in secondary or tertiary care.
