# Supplementary material for: Differences in Pulmonary and Extra-Pulmonary Traits between Women and Men with Chronic Obstructive Pulmonary Disease
Source: J Clin Med. 2022 Jun 26;11(13):3680. doi: 10.3390/jcm11133680 (PMC9267757; doi:10.3390/jcm11133680)
Supplement: Supplementary file 1 [file jcm-11-03680-s001.zip › jcm-1742283-supplementary.pdf]

Supplementary Material

Differences in Pulmonary and Extra-Pulmonary Traits between Women and Men with Chronic Obstructive Pulmonary Disease

Sara Souto-Miranda [1,2,3,*], Alex J. van 't Hul [4], Anouk W. Vaes [1], Jeanine C. Antons [4], Remco S. Djamin [5], Daisy J. A. Janssen [1,6], Frits M. E. Franssen [1,3], Alda Marques [2] and Martijn A. Spruit [1,3]

[1] Department of Research and Development, Ciro, 6085 NM Horn, The Netherlands; anoukvaes@ciro-horn.nl (A.W.V.); daisyjanssen@ciro-horn.nl (D.J.A.J.); frits.franssen@ciro-horn.nl (F.M.E.F.); martijnspruit@ciro-horn.nl (M.A.S.)

[2] Respiratory Research and Rehabilitation Laboratory (Lab3R) and Institute of Biomedicine (iBiMED), University of Aveiro, 3810-193 Aveiro, Portugal; amarques@ua.pt

[3] Department of Respiratory Medicine, Maastricht University Medical Centre, NUTRIM School of Nutrition and Translational Research in Metabolism, Faculty of Health, Medicine and Life Sciences, Maastricht University, 6229 ER Maastricht, The Netherlands

[4] Department of Respiratory Diseases, Radboud Institute for Health Sciences, Radboud University Medical Center, 6525 GA Nijmegen, The Netherlands; alex.vanthul@radboudumc.nl (A.J.v.'t.H.); jeanine.antons@radboudumc.nl (J.C.A.)

[5] Department of Respiratory Diseases, Amphia Hospital, 4818 CK Breda, The Netherlands; rdjamin@amphia.nl

[6] Department of Health Services Research, Care and Public Health Research Institute, Faculty of Health Medicine and Life Sciences, Maastricht University, 6226 NB Maastricht, The Netherlands

* Correspondence: sara.souto@ua.pt.

People referred to secondary care had their first-ever outpatient consultation with a pulmonologist, and therefore before entering the study only had appointments with their family physician.

The tertiary care setting, and specifically Ciro is a multidisciplinary and specialised centre where patients perform a 3-day comprehensive assessment which is followed by the activation of the most suitable treatments (e.g., pulmonary rehabilitation). Since this setting is highly specialised and receives more complex patients (advanced stages of the disease), a larger number of healthcare professionals are involved and therefore more evaluations are conducted. Indeed, these patients can only be referred to Ciro by a pulmonologist. Differences in the assessments between settings are presented in Table S1.

Table S1. Differences in data collected from patients with chronic obstructive pulmonary disease in secondary or tertiary care.

Domain	Measurement	Secondary care	Tertiary care
Sociodemographic data and clinical history	Age	✓	✓
	Sex	✓	✓
	Smoking history	✓	✓
	LTOT	N.A.	✓
	Use of walking aid	N.A.	✓
	Number of exacerbations last 12 months	✓	✓
	Number of hospitalisations last 12 months	✓	✓
Body composition	BMI	✓	✓
	Waist circumference	✓	
	Fat mass	N.A.	✓
	Bone mass content	N.A.	✓
	FFM	N.A.	✓
	Lean mass index	N.A.	✓
Lung function	Spirometry	✓	✓
	Whole-body plethysmography	N.A.	✓
	DLCO	N.A.	✓
	Pimax	N.A.	✓
Physical fitness	6MWT	✓	✓
	CPET	N.A.	✓
	CWRT	N.A.	✓
	1-RM	N.A.	✓
Physical activity	Number of steps	✓	N.A.
Symptoms	mMRC	✓	✓
	HADS	N.A.	✓
	CIS-F	✓	N.A.
Health status	CCQ	✓	N.A.

CAT	N.A.	✓

N.A.: Not assessed. LTOT: Long-term oxygen therapy; BMI: Body mass index; FFM: Fat free mass; DLCO: Diffusion capacity for carbon monoxide; Pimax: Maximum inspiratory mouth pressure; 6MWT: Six-minute walking test; CPET: Cardiopulmonary exercise testing; CWRT: Constant work rate test; 1-RM: 1 maximum repetition; mMRC: Modified medical research council dyspnoea scale; HADS: The hospital anxiety and depression scale; CIS-F: Checklist of individual strength – fatigue subscale; CCQ: Clinical COPD questionnaire; CAT: COPD assessment test. Fat mass, FFM and Lean mass index were assessed through dual-energy X-ray absorptiometry (DEXA), and number of steps was assessed through uniaxial (Digiwalker SW-200; Yamax Corporation, Tokyo, Japan) or a triaxial accelerometer (DynaPort MoveMonitor, McRoberts, The Hague, The Netherlands).
